# Nanometric self-assembling peptide layers maintain adult hepatocyte phenotype in sandwich cultures

**DOI:** 10.1186/1477-3155-8-29

**Published:** 2010-12-12

**Authors:** Jonathan Wu, Núria Marí-Buyé, Teresa Fernández Muiños, Salvador Borrós, Pietro Favia, Carlos E Semino

**Affiliations:** 1 Center for Biomedical Engineering, Massachusetts Institute of Technology, Boston, MA, USA; 2 Department of Bioengineering, Tissue Engineering Laboratory, IQS-Universidad Ramon Llull, Barcelona, Spain; 3 Grup d'Enginyeria de Materials, IQS-Universidad Ramon Llull, Barcelona, Spain; 4 Department of Chemistry, University of Bari, Italy; 5 Translational Centre for Regenerative Medicine (TRM-Leipzig), Universität Leipzig, Leipzig, Germany

## Abstract

**Background:**

Isolated hepatocytes removed from their microenvironment soon lose their hepatospecific functions when cultured. Normally hepatocytes are commonly maintained under limited culture medium supply as well as scaffold thickness. Thus, the cells are forced into metabolic stress that degenerate liver specific functions. This study aims to improve hepatospecific activity by creating a platform based on classical collagen sandwich cultures.

**Results:**

The modified sandwich cultures replace collagen with self-assembling peptide, RAD16-I, combined with functional peptide motifs such as the integrin-binding sequence RGD and the laminin receptor binding sequence YIG to create a cell-instructive scaffold. In this work, we show that a plasma-deposited coating can be used to obtain a peptide layer thickness in the nanometric range, which in combination with the incorporation of functional peptide motifs have a positive effect on the expression of adult hepatocyte markers including albumin, CYP3A2 and HNF4-alpha.

**Conclusions:**

This study demonstrates the capacity of sandwich cultures with modified instructive self-assembling peptides to promote cell-matrix interaction and the importance of thinner scaffold layers to overcome mass transfer problems. We believe that this bioengineered platform improves the existing hepatocyte culture methods to be used for predictive toxicology and eventually for hepatic assist technologies and future artificial organs.

## Background

The liver is an important and complex organ that plays a vital role in metabolism and is responsible for many important functions of the body including glycogen storage, plasma protein production, drug detoxification and xenobiotics metabolization. Due to the importance of this organ in many of the body's daily processes, liver malfunction often leads to death. Most of the activity of the liver can be attributed to hepatocytes, which make up 60-80% of the cytoplasmic mass of the liver [1,2]. Loss of hepatocyte function can result in acute or chronic liver disease and, as a result, substantially compromise the rest of the organ and the body. Many previous strategies have been implemented to maintain these hepatocyte functions *in vitro*, including the use of extracellular matrices such as the current standard, collagen [3-6], Matrigel [7] or liver derived basement membrane matrix [8]. However, the liver carries out and regulates numerous biochemical reactions that require the combined effort of specialized cells and tissues. As a result, isolated hepatocytes removed from their microenvironment soon lose their hepatospecific functions. Therefore, it is important for *in vitro *cultures to provide a system that closely simulates the local environment of an intact liver. Hepatocyte morphology is known to be closely linked to the functional output of the cells [9,10]. Standard cell cultures that seed cells on top of a monolayer of extracellular matrix have been used in the past to successfully culture hepatocytes; however, in certain instances hepatocellular functions become compromised because the cell no longer resembles a natural hepatocyte from a live liver. In many cases, specific cellular phenotypes are directly related to the cellular functions including cell survival, proliferation, differentiation, motility and gene expression [11,12]. Morphogenesis and assembly have been well established to be pertinent in the functional performance of liver-derived cells *in vitro *[10,13-15].

The double-gel "sandwich" method has been shown to improve morphology by embedding the cells between two layers to resemble *in vivo *conditions. Typically, one layer is set on the bottom of a culture dish and an additional layer is placed on top of the hepatocyte monolayer [4,16,17]. Under these conditions, hepatocytes have been shown to maintain some function and differentiation for up to several weeks. Verification of hepatocyte function was shown by specific mRNA [5,18] and protein secretion into culture media [16,19].

The highly oxygen-demanding hepatocytes are commonly maintained in Petri dishes under oxygen-deficient culture conditions and, thus, the cells are forced into anaerobic metabolic states [20]. Hence, oxygen supply in primary hepatocyte cultures is a crucial issue to be addressed. Generally, in cultures in Petri dishes oxygen consumption is no longer dependent upon hepatocellular uptake rates but it is limited by culture medium thickness as well as ambient oxygen concentrations. However, regardless of these constraints, hepatocytes are able to tolerate the hypoxic conditions by satisfying energy requirements through anaerobic glycolysis [20]. In any case, a previous study has shown that hepatospecific functions are oxygen-dependent, especially demonstrated in the poor production of albumin, urea and drug metabolites over a 14-day study period in common Petri dish models compared to enhanced oxygen delivery cultures on gas-permeable films [21]. Furthermore, it was shown as early as in 1968 that commonly used medium depths of 2-5 mm in Petri dishes rapidly produced hypoxic conditions when hepatocytes respired at their physiological rate [22]. Therefore, because plastic walls and culture medium are efficient barriers of oxygen diffusion, it is important to create a system in which a physiological oxygen supply is maintained [23,24].

More recently, the use of self-assembling peptides has been implemented and verified to be an excellent scaffold for cell culture [25-30]. Especially, RAD16-I (Table 1) has been extensively used in most of the studies. Not only does it provide an excellent three-dimensional microenvironment, but also it allows for the design and preparation of a tailor-made scaffold. This represents a novel approach to tissue engineering, which traditionally has relied on materials that were unknown in composition, like Matrigel, or not possible to design and alter, such as collagens. Furthermore, the versatility of the modification of this material allows for the introduction of functionalized peptide motifs, such as the signaling sequence GRGDSP (RGD) from collagen and YIGSR (YIG) from laminin [27,31], which target an integrin receptor and the 67 kDa laminin receptor, respectively [32]. Those motifs have been shown to be crucial in the activation of numerous vital cell functions including migration, proliferation, and cell attachment [33,34]. In one study, grafted adhesion peptides RGD and YIG were proved to promote hepatocyte adhesion to the surface by 60% [35]. Also, RGD-containing synthetic peptides coated on plastics promoted hepatocyte adhesion and differentiated function [36]. Recently, we combined RAD16-I with modified self-assembling peptides containing the integrin-binding sequence RGD, the laminin receptor binding sequence YIG and the heparin binding sequence present in collagen IV TAGSCLRKFSTM (TAG), in order to obtain a functionalized matrix scaffold [31]. We analyzed several liver-specific functions in terms of gene expression by means of quantitative PCR of albumin, hepatocytes nuclear factor 4-alpha (HNF4-alpha), multi-drug resistant protein 2 (MDR2) and tyrosine aminotransferase (TAT). When we compared two sandwich dimensions with layers of 1 mm and 0.5 mm, we observed, as expected, that the thinner configuration promoted upregulation of some specific genes due to the improvement of gas, nutrient and toxin exchange. However, when we analyzed expression of oxidative enzymes, in particular the cytochrome P450 3A2 (CYP3A2), the expression of the enzyme was downregulated at the same levels of the standard collagen sandwich cultures for all the conditions tested [31]. In a recent work, Wang *et al*. cultured freshly isolated rat hepatocytes over surfaces of self-assembling peptide gels, which improved many adult hepatic functions as compared to the double collagen layer or collagen sandwich culture [37]. In this type of surface, hepatocytes cultures developed into spheroids, easily to handle and with good hepatic performance. Nevertheless, this culture system does not allow an intimate interaction of the hepatocytes with the matrix. Moreover, a platform that uses a synthetic gel material in a sandwich configuration enables to rationally functionalize the matrix and thus to obtain specific cell responses.

**Table 1:** Self-assembling peptide sequences

Sequence Name	Peptide Sequence	Function
RAD16-I	AcN-RADARADARADARADA-CONH_2 _	Base Sequence

RGD	AcN-G**RGD**SPGGRADARADARADARADA-CONH_2 _	Integrin Binding

YIG	AcN-**YIG**SRGGRADARADARADARADA-CONH_2 _	Laminin Binding

Functional peptide motifs, denoted in bold, are inserted onto the N-terminal of the base sequence of RAD16-I. (R = Arginine; A = Alanine; D = Aspartic Acid; G = Glycine; Y = Tyrosine; I = Isoleucine; S = Serine)

Since the 70's in microelectronics, non equilibrium, cold, gas plasmas are effective methods utilized in material science and technology, including biomaterials, to tailor surface composition and materials properties. Plasma etching, plasma enhanced chemical vapor deposition (PECVD) and grafting of chemical functionalities by plasma are the three main surface modification processes. Appealing features of plasma techniques are the following: they work at room temperature; modifications are limited within the topmost hundreds nanometers of the materials, with no change of the bulk; use of very low quantities of gas/vapor reagents; no use of solvents; easy integration in industrial process lines [38]. Cold plasmas are used to tailor surface properties of materials intended to be used in biomedical applications. Due to their ability of tuning independently surface chemical composition and topography (e.g., roughness, patterns, etc.), plasma treatments allow processes like: the synthesis of non-fouling coatings, capable of discouraging the adhesion of proteins and cells at the biomaterial surface [39,40]; the optimization of the adhesion and behaviour of cells onto biomaterials [41-43] and membranes [44,45]; and the functionalization of surfaces for covalent immobilization of biomolecules like peptides [46] and saccharides [47,48] to mimic the extracellular matrix. One example are the plasma-deposited acrylic acid (PdAA) coatings [49], which are used in the biomedical field to provide the surface of biomaterials with -COOH groups for improving cell adhesion and growth [50-52] or for further immobilization of biomolecules [46-48]. Also, surfaces modified with pentafluorophenyl methacylate (PFM) have been successfully used to anchor biologically active motifs, since this monomer easily reacts with molecules containing primary amines, such as bioactive peptides [53,54].

Studies have tried cocultures of hepatocytes with other cells such as fibroblasts with the idea that nonparenchymal cell factors may promote and induce specific hepatocyte expression [55,56]. Others have tried to achieve *in vivo *level induction by focusing on culture substratum using complex matrices including fibronectin [57], extracts from liver [58] and Matrigel [59]. Currently, the best culture conditions for preserving primary hepatocytes are still unresolved. Therefore, in this work we develop a new platform where the hydrogel scaffold dimensions can be several orders of magnitude smaller (from 500 μm down to nanometric scale). Our strategy to control the peptide layer dimensions within a nanometric scale made possible to maintain the CYP3A2 activity for long periods in rat hepatocyte cultures. Briefly, in order to build our new biomaterial platform, we used two biocompatible porous membranes as main structural support for the hydrogel: PEEK-WC-PU, (poly(oxa-1,4-phenylene-oxo-1,4-phenylene-oxa-1,4-phenylene-3,3-(isobenzofurane-1,3-dihydro-1-oxo)-diyl-1,4-phenylene) modified with aliphatic polyurethane) [60] and PTFE (polytetrafluorethylene). These biocompatible membranes were chemically modified by means of two different plasma modifications in order to immobilize RAD16-I peptides. The anchored RAD16-I molecules directed the self-assembling of additional soluble RAD16-I peptides, which assemble forming a thin scaffold layer. Finally, we were able to obtain expression levels of albumin, CYP3A2 and HNF4-alpha similar to fresh hepatocytes by using the membranes with the controlled self-assembling peptide layer in a sandwich culture system during seven days.

## Results and discussion

In this work, we attempt to address the concerns of traditional hepatocyte culture methods by combining tissue engineering technologies. Our sandwich culture method is adjusted from the traditional double gel layer "sandwich" technique to address diffusion issues. Instead of culturing the hepatocytes under a thick second layer of peptide, the cells are entrapped under a biocompatible porous membrane (PEEK-WC-PU or PTFE), previously modified through plasma processes to allow dimensional control of a thin hydrogel-coating layer. This self-assembling peptide layer contains signaling peptide sequences to promote specific cell responses, mimicking the cell-matrix interactions that are lost in isolated hepatocytes.

### Dimensional control of self-assembling peptide layer

**Figure 1:**
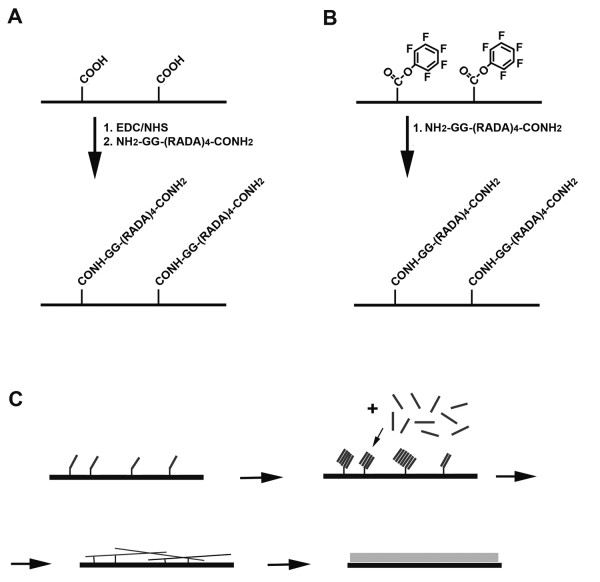
**Development of nanometric self-assembling peptide layers on thin porous membranes**. (**A**) Plasma deposition of acrylic acid onto membrane surfaces. RAD16-I peptide sequences are immobilized to the deposited -COOH. (**B**) Plasma deposition of pentafluorophenyl methacrylate (PFM) onto membrane surfaces. RAD16-I peptide sequences are immobilized to the deposited PFM. (**C**) Model describing the formation of a thin layer of self-assembling peptide gel on a membrane substrate using immobilized self-assembling peptides as attachment points.

**Figure 2:**
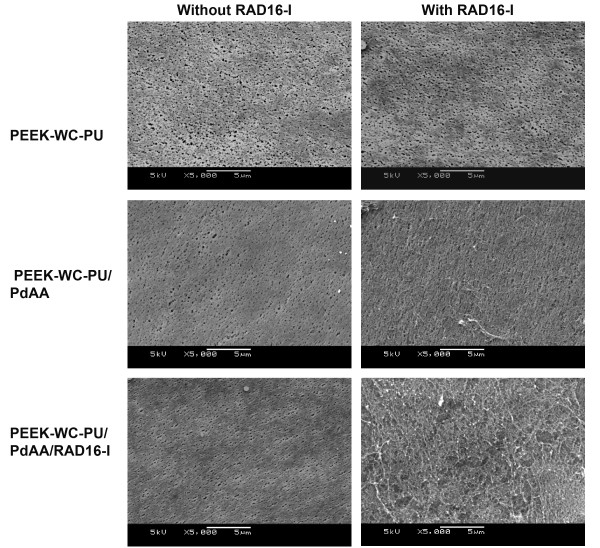
**Self-assembling nanofiber network development on PEEK-WU-PC membranes**. SEM images of fiber formation of RAD16-I self-assembling peptide on unmodified PEEK-WC-PU membranes (top row), plasma-deposited acrylic acid PEEK-WC-PU membranes, PEEK-WC-PU/PdAA (middle row), and plasma-modified RAD16-immobilized PEEK-WC-PU membranes, PEEK-WC-PU/PdAA/RAD16-I (bottom row), after incubation in absence (left column) or presence (right column) of soluble RAD16-I peptide.

In this work, membrane surfaces were modified by two non-equilibrium plasma processes: plasma enhanced chemical vapor deposition (PECVD) and plasma grafting (PG). Plasma treatments can be used to tune surface properties, including electric charge, wettability, free energy, surface chemistry and morphology. This ability to optimize surface conditions can affect cellular behavior and attachment either directly, for instance, through guided cell spreading or indirectly, for example, through controlled protein adsorption on the surface. The more recent and advanced uses in plasma treatments involve the immobilization of biomolecules onto biomaterial surfaces to promote specific cellular responses at the molecular and cellular levels [47,54,60]. In this study, membranes were modified by plasma deposition of acrylic acid (hereby abbreviated as "PdAA") or by plasma grafting of pentafluorophenyl methacrylate, PFM (hereby abbreviated as "PgPFM"). In the first case, the monomer was subjected to plasma and then polymerized on the surface whereas in the second case, the surface was activated by plasma creating active groups that react with the oncoming monomer. Both modifications would allow the posterior attachment of a peptide to the surface. Therefore, we developed a method based on two simple steps: 1, RAD16-I self-assembling peptides containing a free amino termini group (NH_2_-RAD16-I) were immobilized on the surface of a porous membranes (Figure 1A and 1B) and 2, then RAD16-I peptide solution (1% (w/v)) was incubated over the peptide-immobilized membranes, followed by a water rinse to remove unbound and unassembled peptides (Figure 1C). The attached peptide, with the same aminoacid sequence as RAD16-I, acted as an anchor to stabilize the self-assembled nanofibers formed from the RAD16-I peptide solution. Peptide attachment to the membranes (PEEK or PTFE) was confirmed by x-ray photoelectron spectroscopy (XPS) and by detection of fluorescein-conjugated peptides (data not shown).

**Figure 3:**
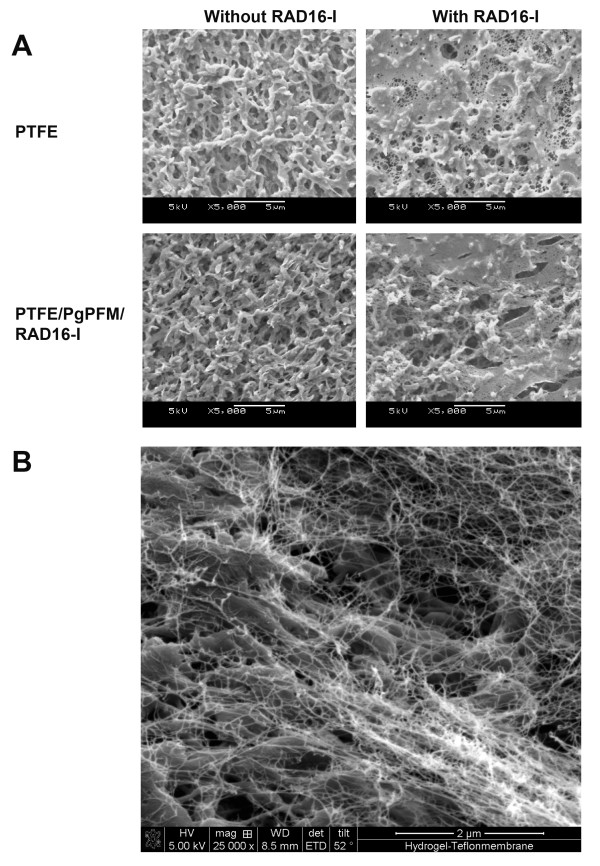
**Self-assembling nanofiber network development on PTFE porous membranes**. (**A**) SEM images of fiber formation of RAD16-I self-assembling peptide on unmodified PTFE membranes (top row) and plasma modified RAD16-I immobilized PTFE membranes, PTFE/PdPFM/RAD16-I membranes (bottom row) after incubation in absence (left column) or presence (right column) of soluble RAD16-I peptide. (**B**) Close up of a SEM image of a PTFE/PdPFM/RAD16-I membrane after incubation in presence of of soluble RAD16-I peptide.

SEM was used to evaluate the formation of the hydrogel layer on the membranes. As expected at this magnification, alterations due to plasma treatment or RAD16-I peptide immobilization were not visibly apparent (Figure 2 and 3). In the case of PEEK-WC-PU membranes modified with the RAD16-I peptide (PEEK-WC-PU/PdAA/RAD16-I), the native membranes (PEEK-WC-PU) and the acrylic acid modified membranes (PEEK-WC-PU/PdAA) were used as controls. After one-hour incubation with the self-assembling peptide solution at 1% (w/v), followed by water rinsing, the native PEEK-WC-PU membrane showed no fiber formation and the PEEK-WC-PU/PdAA membrane displayed some non-homogeneous peptide fiber attachment (Figure 2). Interestingly, the PEEK-WC-PU/PdAA/RAD16-I membrane demonstrated the best fiber formation of the three conditions (Figure 2). The peptide layer was both thin and homogenous, creating a nanometric mesh, which seemed not to obstruct the pores of the native membrane beneath. On the other hand, self-assembling nanofiber formation was also observed on the Biopore PTFE membranes (Figure 3A). In this case, only the native PTFE membrane was used to compare against the peptide-modified PTFE (PTFE/PgPFM/RAD16-I). Surprisingly, after the one-hour incubation and rinsing, both the native and modified PTFE membranes presented the same fiber formation pattern of self-assembling peptide. The peptides seemed to have assembled into a very thin web layer using the protruding features of the membrane. Closer examination revealed a mesh of individual fibers in the membrane pores (Figure 3B).

### Hepatocyte attachment on thin hydrogel layer

**Figure 4:**
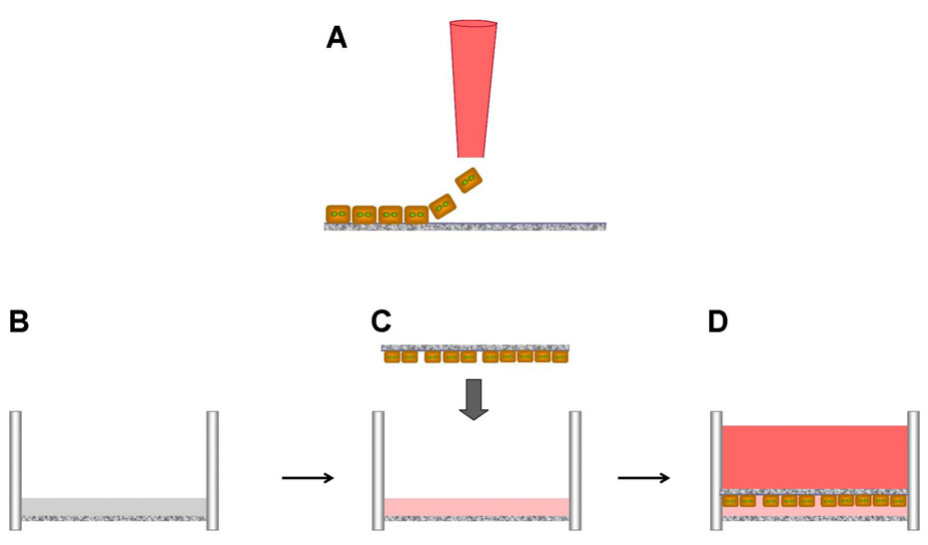
**Self-assembling peptide-coated membranes seeded with hepatocytes**. (**A**) Hepatocytes are loaded on top of a thin layer of self-assembling peptide gel on a membrane substrate described in Figure 1. (**B**) A tissue culture insert is coated with a layer of ~0.5 mm of self-assembling peptides. (**C) **Gel formation is induced by addition of media, and the inverted cell-seeded membrane from **A **is placed on top of the equilibrated gel. (**D**) Finally, the sandwich is covered with media. Therefore, the new sandwich culture system consist of a hydrogel layer at the bottom (~0.5 mm) covered by a thin layer of self-assembling peptide-coated on a porous membrane (PEEK or PTFE). The hepatocytes are placed in within both layers.

**Figure 5:**
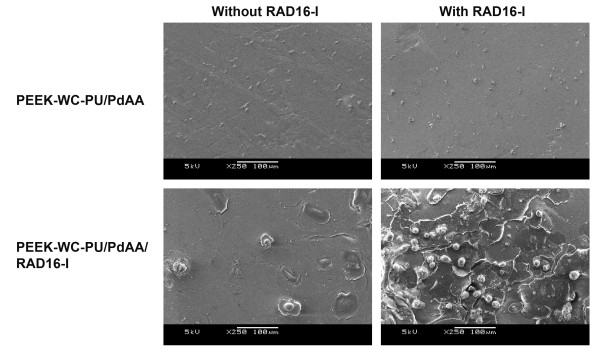
**Hepatocyte attachment on a self-assembling peptide covered PEEK-WC-PU porous membrane**. SEM images of hepatocyte attachment with (left column) and without (right column) RAD16-I incubation on plasma-deposited acrylic acid (top row, PEEK-WC-PU/PdAA) and plasma-modified RAD16-immobilized (bottom row, PEEK-WC-PU/PdAA/RAD16-I) PEEK-WC-PU membranes.

**Figure 6:**
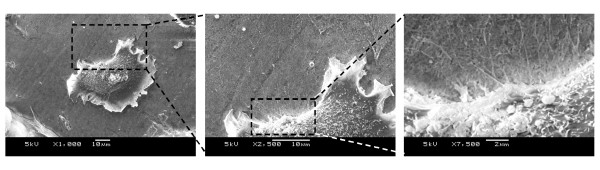
**Close-up images of a hepatocyte attached on a self-assembling peptide covered PEEK-WU-PC porous membrane**. SEM images of a single hepatocyte on PEEK-WC-PU/PdAA/RAD16-I+RAD16-I. At closer magnifications, cytoplasmic projections seem to adhere to the self-assembling peptide substrate (from left to right).

The next objective was to assess the attachment of hepatocytes onto the self-assembling peptide-coated modified membranes. To determine whether cellular attachment was specifically enhanced by the presence of the self-assembling peptide layer, the hepatocytes were incubated for 8 hours and then the media was changed in order to remove dead cells (Figure 4A). After 24 hours post cell-seeding the PEEK-WC-PU/PdAA did not bind any cells, as expected (Figure 5). Likewise, there was no cellular attachment apparent on the PEEK-WC-PU/PdAA that was previously incubated with soluble peptide, which yielded a patchy, variable, and unreliable fiber formation (Figure 2). On the other hand, the PEEK-WC-PU/PdAA/RAD16-I membranes demonstrated cell binding in both conditions (Figure 5). Without the peptide incubation, a few cells unexpectedly still attached to the surface. There was no fiber matrix present, however, the immobilized RAD16-I peptides might have provided a more favorable cell-attaching surface than the PEEK-WC-PU/PdAA substrate. Finally, with the peptide incubation, the surface was completely filled with hepatocytes. It is apparent that the self-assembling peptide fiber network vastly enhanced hepatocyte attachment. A close-up image of one of the hepatocytes reveals an intricate cellular attachment with the substrate (Figure 6).

**Figure 7:**
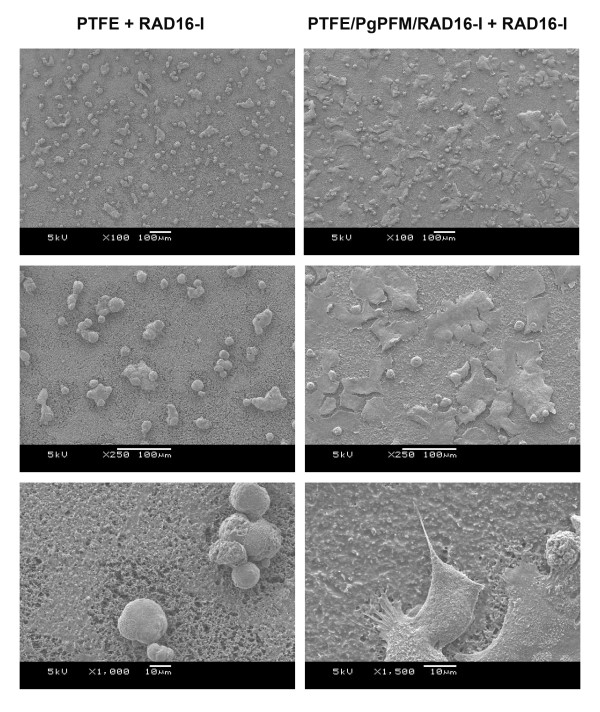
**Hepatocyte attachment on a self-assembling peptide covered PTFE porous membrane**. SEM images of hepatocyte attachment with RAD16-I incubation on native PTFE (left column, PTFE + RAD16-I) and plasma-grafted PFM RAD16-immobilized PTFE membranes (right column, PTFE/PgPFM/RAD16-I + RAD16-I). Note: SEM image of hepatocyte attachment on native PTFE membrane. Hepatocytes appear to pull off surrounding peptide without the anchorage of immobilized peptides and form clusters. The cells are unable to interact with the rigid substrate beneath the peptide and, thus, do not achieve a flat morphology (see bottom left panel). Instead, hepatocyte attachment on PFM RAD16-I-immobilized PTFE membranes ends in the formation of cytoplasmic projections visibly adhere to the self-assembling fibers.

On the other hand, the native (PTFE) and peptide-modified (PTFE/pgPFM/RAD16-I) membranes, both incubated with soluble RAD16-I, supported hepatocyte attachment (Figure 7). Interestingly, the morphology of the cells for each of the membranes was very different. For instance, on the native membrane, the hepatocytes remained round and spherical throughout the entire surface. Likewise, the cells tended to clump and form spheroids. On the other hand, the peptide-modified membrane mainly contained cells with a flat and extended morphology (Figure 7). The cells on this membrane tended not to cluster and form spheroids. The spread and extended morphology is more favorable for hepatocytes to develop cell-matrix and cell-cell interactions. For example, this morphology could promote polarization and the formation of bile canilicular spaces between neighboring cells. Although both membranes were visibly identical, we propose that the immobilized RAD16-I created an anchor for the peptide layer on the peptide-modified PTFE and thus generated a stronger interaction between the nanofiber coating and the membrane. We speculate that cell-matrix interaction was more stable in the peptide-modified membranes than in the native one promoting the development of a flat and extended morphology. When nanofibers were not immobilized, the cells appear to pull off surrounding unanchored peptide without being able to interact with the membrane, and instead interacting with surrounding cells to form clusters.

### Modified Sandwich Culture of Primary Hepatocytes

After demonstrating that our substrates were able to promote cell attachment and proper morphology, the following objective was to determine to what extent the self-assembling peptides enhanced hepatocellular function, especially CYP3A2 expression. In a recent publication, we observed that using self-assembling peptide sandwich with layer dimensions between 0.5-1.0 mm, the expression of oxidative enzymes, in particular CYP3A2, in all the conditions tested was highly downregulated [31].

**Figure 8:**
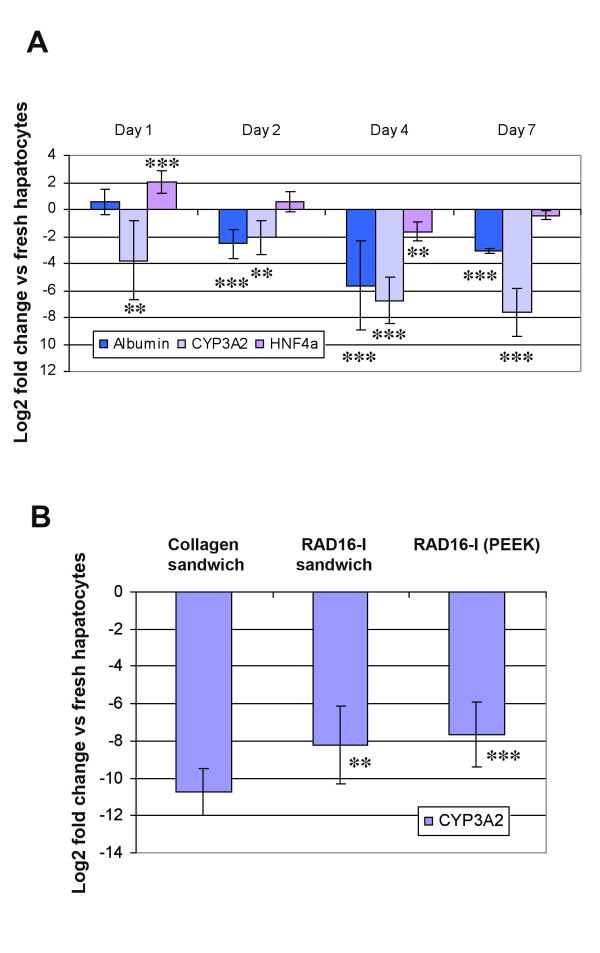
**Expression of hepatocyte markers of cells in sandwich cultures of self-assembling peptide scaffolds and PEEK-WC-PU membranes**. (**A**) Gene expression profile of albumin, CYP3A2, and HNF4-alpha obtained by quantitative PCR relative to gene expression in freshly isolated hepatocytes. Cells cultured on modified PEEK-WC-PU membranes incubated with RAD16-I. (**B**) Comparison of CYP3A2 gene expression relative to freshly isolated hepatocytes by quantitative PCR with previous results at 7 days of collagen cultures (collagen sandwich) are compared with both self-assembling peptide RAD16-I sandwich cultures (RAD16-I sandwich) and sandwich cultures of self-assembling peptides RAD16-I and PEEK-WC-PU membranes (RAD16-I (PEEK)). Data in **A **and **B **is presented as mean ± SD (with statistical significances indicated as ** for p < 0.01 and *** for p < 0.001).

Thus, modified peptide sandwich cultures were prepared similar to typical sandwich cultures except for the top layer of soluble peptide that was substituted with the inverted cell-seeded modified membrane (Figure 4). Cultures were observed over a week-long period and quantitative PCR (qPCR) was performed to measure hepatospecific biomarkers expressed in fresh hepatocytes. Gene expression profile of albumin, CYP3A2, and HNF4-alpha relative to gene expression in freshly isolated hepatocytes over a period of seven days was initially performed using modified sandwich cultures with PEEK-WC-PU membranes (Figure 8). Results were attained in three separate experiments presented on a log base 2 scale. Therefore, a 2-fold upregulation is equivalent to 4 fold (= 2^2^) increased expression. In addition, values between -1 and +1 are considered equivalent to fresh hepatocyte levels.

After 24 hours post-seeding, the cells expressed great levels of albumin and HNF4-alpha (Figure 8A). Albumin expression was close to fresh levels at day 1, then began to slightly decline until day 4 and by day 7, appeared to have improved to -3-fold downregulation. On the other hand, HNF4-alpha expression maintained within a close range to fresh cell levels. CYP3A2 was downregulated at day 1 and slightly evened off around a -7-fold after a week. However, our system at this point is still about 1.5-fold better than the current gold standard method of culturing hepatocytes with collagen or double gel layers of RAD16-I self-assembling peptides (Figure 8B).

Then, in order to see if PTFE membranes were able to increase the expression profile of CYP3A2 due to its bigger pore size and as consequence, possible improvement of mass transfer issues, gene expression relative to freshly isolated hepatocytes over a period of seven days -for modified sandwich cultures using peptide-modified PTFE membranes- was also monitored. In addition we decided to study the effect that functionalized nanofiber network -with biological active motifs- could have on specific cell-receptor signals and therefore improving hepatocyte phenotype. Thus, the nanofiber layer was prepared by using 100% of RAD16-I peptide or blended with small percentages of functionalized ones (RGD and YIG peptides) carrying receptor-binding sequences (Table 1). Different layer compositions were tested: RAD16-I, 5% RGD in RAD16-I, 5% YIG in RAD16-I, and a mix of 2.5% RGD and 2.5% YIG in RAD16-I. RNA samples were collected at day 7 to determine if the peptide layer and the functional motifs enhanced CYP3A2 expression.

**Figure 9:**
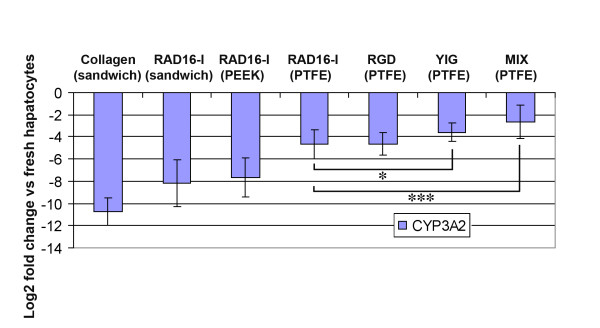
**Expression of CYP3A2 of in sandwich cultures of self-assembling peptide scaffolds and PTFE membranes**. Comparison of CYP3A2 gene expression relative to freshly isolated hepatocytes at day 7 by quantitative PCR with previous results from Collagen sandwiches and RAD16-I sandwiches [31], and the performed in this manuscript with PEEK-WC-PU membranes and PTFE membranes. Particularly, cultures YIG (PTFE) and MIX (PTFE) are significantly different from RAD16-I (PTFE) with p < 0.05 and p < 0.001, respectively. Data is presented as mean ± SD (with statistical significances indicated as * for p < 0.05 and *** for p < 0.001).

Interestingly, CYP3A2 expression in RAD16-I-modified PTFE membranes increased dramatically over 7 days in culture compared to the previous RAD16-I-PEEK-WC-PU membranes (Figure 9). Although, RGD PTFE membranes (5% RGD/95% RAD16-I) were similar to RAD16-I a good improvement in CYP3A2 expression was observed with YIG PTFE ones (5% YIG/95% RAD16-I) and even better with the MIX PTFE membranes (2.5% RGD/2.5% YIG/95% RAD16-I), which provided a vast improvement over the RAD16 PTFE condition (Figure 9). Again, compared to the data from the PEEK-WC-PU membranes, the day 7 CYP3A2 levels of all conditions on the PTFE membranes are significantly better (Figure 9). With respect to the previous PEEK-WC-PU material, the RAD16-I condition on PTFE provided a 3-fold improvement. This was about an x8 (= 2^3^) increase in gene expression. We conjecture this enhancement to be due to physical differences between membrane geometry and topography. As seen in the SEM images (see Figure 2 and 3), the porosity of the PTFE membrane was much higher and the pore sizes were much larger compared to PEEK ones. Tying back to our hypothesis of culture improvement by reducing oxygen and nutrient diffusion barriers, the PTFE membranes provide much less of an obstruction and facilitate better exchange with the microenvironment. In comparison to the other PTFE conditions, the RAD16-I and RGD cultures appear to be on par with each other. The YIG condition slightly improves upon the previous two. Likewise, the mix condition of RGD and YIG furthermore enhances the CYP3A2 gene expression (at mRNA levels) to almost -2.5-fold. This clearly indicates the positive effect on CYP3A2 increase caused by the presence of the bioactive motifs YIG alone or the mix YIG+RGD. This is a tremendous improvement over the standard collagen method that produced about a -10.5-fold downregulation. This 8-fold difference translates to a 256-fold (= 2^8^) improvement on gene expression at the level of mRNA in the MIX modified sandwich system as compared to the typical collagen sandwich culture (Figure 9).

## Conclusions

We have successfully shown that our novel bioengineering platform can maintain expression levels of albumin, CYP3A2 and HNF4-alpha similar to fresh hepatocytes for as long as a week. This was ultimately done by improving the biophysical features of traditional sandwich cultures by optimizing the top peptide layer dimension to orders of nanometers to facilitate oxygen exchange and nutrient diffusion. Additionally, the biochemical aspects of typical cultures were enhanced by engineering the scaffold with the introduction of functional peptide motifs to strategically target certain cell receptors responsible for the activation of numerous vital cell functions. We believe that our new platform has improved significantly the existing culture methods, opening a new possibility for the pharmacological industry.

## Methods

### Hepatocyte Isolation

Hepatocytes were isolated from male Fisher rats weighing 150-180 g using a modification of the Seglen 2-step collagenase perfusion procedure [61]. Cell yield and viability were determined via trypan blue exclusion and hemocytometry. Typically, 250-300 million hepatocytes were harvested per rat liver with viability ranging from 85-92%. Following isolation, cells were initially suspended in Hepatocyte Culture Medium (HCM, Cambrex, MD, CC-3198), containing 2% fatty acid free BSA (bovine serum albumin), transferrin, insulin, recombinant human EGF (epithelial growth factor), ascorbic acid, hydrocortisone and gentamycin/amphotericin.

### Plasma Modification of Membranes

PEEK-WC-PU membranes were kindly provided by the De Bartolo Lab (Institute on Membrane Technology, National Research Council of Italy). PTFE membranes were purchased (Biopore, BCGM00010).

PEEK-WC-PU membranes were modified with a PE-CVD surface functionalization process fed with acrylic acid (AA) vapors in order to create a thin stable plasma-deposited acrylic acid (pdAA) coating characterized by a certain density of -COOH groups. Membranes were plasma-coated in a radio frequency (13.56 MHz) driven stainless-steel parallel plate plasma reactor [51]. AA vapors were fed from a liquid reservoir kept a room temperature, at a pressure of 0.2 mbar, and samples were exposed at 100 W for 5 min (PEEK-WC-PU/PdAA). Before use AA was degassed with freeze-thaw cycles. The deposition results in 10 ± 1 nm thick coatings characterized by a surface density of about 4% carboxylic groups over all carbon atoms of the coatings, and a O/C atomic ratio of 0.29, as measured by X-rays photoelectron Spectroscopy [51]. PdAA-coated membranes substrates were then immersed in 1-ethyl-3-(3-dimethylamino-propyl) carbodiimide (EDC) and N-hydroxysuccinimide (NHS) in morpholine ethane sulfonate (MES) buffer to activate -COOH groups. Afterwards, membranes were incubated overnight in a water solution of NH_2_-GG-RAD16-I (10 mg/ml) at 37°C to obtain the final PEEK-WC-PU membranes with immobilized RAD16-I (PEEK-WC-PU/PdAA/RAD16-I) (see Figure 1A).

Instead, PTFE membranes were modified by graft polymerization of pentafluorophenyl methacrylate (PFM) in a two-step process. The membranes were placed in a cylindrical Pyrex reactor equipped with a copper coil that generates plasma under vacuum conditions (0.02 mbar). Argon was fed into the chamber increasing the pressure to approximately 0.06 mbar and samples were exposed to argon plasma at 50 W for 5 min. Afterwards, argon inflow was closed and PFM vapor was introduced into the reactor and allowed to polymerize for 1 min (PTFE/PgPFM) without plasma. After treatment, membranes were soaked overnight in an aqueous solution of NH_2_-GG-RAD16-I (10 mg/ml) at 37°C to obtain the final PTFE membranes with immobilized RAD16-I (PTFE/PgPFM/RAD16-I) (see Figure 1B).

### RAD16-I peptide layer membrane coating

All membranes, including the native PEEK-WC-PU, PEEK-WC-PU/PdAA, PEEK-WC-PU/PdAA/RAD16-I, native PTFE, PTFE/PgPFM and PTFE/PgPFM/RAD16-I membranes were sterilized using a 70% ethanol. After the ethanol rinse, the membranes were autoclaved for 20 min at 120°C, followed by a 10 min drying step.

PuraMatrix RAD16-I peptide (BD Biosciences, 354250) was used to coat the surface of the membranes. In cases where modified peptides (RGD or YIG) were included, the modified peptides were blended in a 95:5 proportion with the prototypic peptide RAD16-I (prototypic:modified). A volume of 50 μl of peptide was used to thinly cover the surface of the 0.5 in × 0.5 in square membrane samples. The self-assembling peptide solution becomes a hydrogel through contact with salt-containing buffers or media [62]. However, in order to control the thickness of the peptide layer, gelation was not initialized through the introduction of media, but the soluble peptide was allowed to incubate for an hour to permit any self-assembling to occur with the immobilized peptide strands (Figure 1C). Following the incubation, a rinse step was included that entailed dipping the coated membranes into deionized water ten times in succession to remove any non-assembled peptide.

### Peptide sandwich preparation

In order to seed the cells on the peptide-coated membranes, these were incubated with a volume of hepatocyte cell suspension in HCM at a final density of 65,000 cells/cm^2 ^and left to attach in a 37°C incubator for 8 h (Figure 4A). Following the 8 h attachment period (optimized attachment time), the medium was changed to remove dead cells. Meanwhile, the bottom peptide layer was prepared by loading 0.25 ml of peptide into a Millicell tissue culture insert (Millipore, PICM 03050). Then, 1.5 ml of HCM were added underneath the insert membrane to induce gelation, forming a 1 mm-thick gel (Figure 4B). Following the gelation of the peptide, 0.4 ml of HCM were added into the insert and the gel was allowed to equilibrate for 30 min in an incubator at 37°C. To complete the peptide sandwich, the membrane containing the attached cells was inverted on top of the gel layer in the tissue culture insert (Figure 4C). Then, 0.3 ml of HCM was added to the inside of the insert (Figure 4D). Cultures were maintained in a water-jacketed incubator at 37°C and 5% CO_2_. Media was changed every day so that a fresh reservoir of 1.6 ml surrounded the outside of the insert and 0.3 ml replaced the inside of the insert.

### SEM sample preparation

Membrane samples for all conditions (treated/not treated with soluble peptide, incubated/not incubated with hepatocytes) were submerged for 20 min in a fixative mixture containing 2% glutaraldehyde (Sigma, G7526) + 3% paraformaldehyde (Sigma, P6148) in PBS (Invitrogen, 14040). Following the fixing, a series of ethanol dehydration steps were performed, which included incubating the samples for 15 min in 50% ethanol, then 30 min in 75% ethanol, then 60 min in 90% ethanol, followed by two changes of 100% ethanol, where the samples were kept until the next step. Using a Tousimis Super Critical Point Autosamdri815 Dryer, the ethanol-saturated samples underwent a process in which the ethanol was slowly exchanged with CO_2 _and then dried at the critical pressure and temperature of CO_2_. The dried samples were subsequently sputter-coated in vacuum (Denton Vacuum, LLC) with gold (30 sec, approximate thickness 4-5 nm). SEM was carried out using a JEOL JSM 6060 Scanning Electron Microscope at an accelerating voltage of 5 kV.

### Quantitative reverse transcriptase PCR (qRTPCR)

RNA samples were obtained by treating cells with Trizol Reagent (Invitrogen, 15596-026) and storing samples in a -80°C freezer until use. Samples were homogenized through pipetting with a 20G needle. Chloroform (Mallinckrodt Chemicals, 4440-04) was mixed thoroughly with each sample and then spun to separate the protein and RNA. The protein fractions were individually stored in a -20°C freezer for further assays. The "clear" RNA supernatants of the samples were transferred to separate tubes with equal volumes of 70% ethanol. Using the RNEasy Mini Kit (Qiagen, 74104), the RNA mixture was extracted using a series of reagents and spin cycles. RNA of freshly isolated hepatocytes was extracted and used as a control. The obtained RNA samples were quantified and the quality assessed using a NanoDrop ND-1000 Spectrophotometer. RNA samples of poor quality (260/280 and 260/230 ratios not approximately 2.0) were cleaned up using the Qiagen RNA Clean Up procedure included in the RNEasy Mini Kit. The RNA samples were treated with DNAse Buffer and DNAse I (Invitrogen, 18068) to remove contaminant genomic DNA. An amount of 150 ng of RNA for each sample was reversed transcribed with an Omniscript Reverse Transcription Kit (Qiagen, 205111). EDTA was added to each sample and then incubated at 65°C for 10 min. Next, a master mix of DEPC water, dNTP, Random Hexamers, RNase Inhibitor, RT Buffer and Omniscript RTO Reverse Transcriptase was added to each sample and incubated at 37°C for 1.5 h. Quantitative PCR was performed using QuantiTect SYBR Green PCR kit (Qiagen, 204143) in a MJ Opticon Monitor instrument (Applied Biosystems). PCR primers were designed to obtain 150-200 base pair amplification product. The primer sequences used are shown in Table 2. The PCR protocol entailed: incubation at 95°C for 15 min, followed by 45 cycles of incubating the samples at 94°C for 15s, annealing at 51-55°C for 30s and extending at 72°C for 30s. A melting curve was performed to assess the purity of the products formed. Using the 2^-ΔΔCt ^method, relative gene fold changes were determined compared to the ribosomal unit 18s as a housekeeping gene. Samples were then compared to the gene expression in freshly isolated hepatocytes. P-values of a test of significance were calculated using Excel.

**Table 2:** RT-PCR primers of albumin, HNF4-α, CYP3A2, and the housekeeping gene, 18S

Primer Name	Primer Sequence
Albumin forward	5'-GGTGCAGGAAGTAACAGACTTTG-3'

Albumin reverse	5'-TAACTTGTCTCCGAAGAGAGTGTG-3'

HNF4-α forward	5'-CTGAGACTCCACAGCCATCA-3'

HNF4-α reverse	5'-CTAGATGGCTTCCTGCTTGG-3'

CYP3A2 forward	5'-GTAGTACTCTTTCCATTCCTCACCC-3'

CYP3A2 reverse	5'-GGTGCTTATGCTTAGAATCCAGAC-3'

18s forward	5'-GCAATTATTCCCCATGAACG-3'

18s reverse	5'-GGCCTCACTAAACCATCCAA-3'

## Competing interests

The authors declare that they have no competing interests.

## Authors' contributions

JB and NMB developed the nanometric self-assembling peptide layers on membranes, and contributed to electron microscopy techniques and data analysis. JB carried out hepatocyte culture experiments and gene expression technique. NMB designed and prepared the PFM-functionalized membranes and RAD16-I peptide anchorage. TFM contributed to gene expression experiments and data analysis. SB participated in the design of the PFM-functionalized membranes. PF developed AA-modified membranes as well as RAD16-I peptide immobilization. CES conceived of the study, and contributed in its design and coordination. JB, NMB and CES wrote the paper. All authors read and approved the final manuscript.
